# Replacing HaCaT keratinocytes with 3D reconstructed human epidermis in a THP-1 coculture dendritic cell activation assay: quantitative skin sensitization potency assessment and structure–potency relationships

**DOI:** 10.1016/j.namjnl.2026.100137

**Published:** 2026-09-26

**Authors:** Brunhilde Blömeke, Niklas Peter, Udo Bock, Boris Peter Müller, Peter Griem, Rüdiger Wittlake, Johannes Panten

**Affiliations:** a Department of Environmental Toxicology, Trier University, Trier, Germany; b Symrise AG, Mühlenfeldstraße 1, Holzminden, Germany

**Keywords:** Skin sensitization, Reconstructed human epidermis (RHE), THP-1 cells, Quantitative potency assessment, Applicability domain

## Abstract

Reliable assessment of skin sensitization hazard and potency remains challenging for fragrance and other substances. Although current in chemico and in vitro assays perform well within their respective applicability domains, challenges remain for highly lipophilic, poorly water-soluble, cytotoxic chemicals and substances requiring metabolic or abiotic activation. To address these challenges, the established 2D Cocultured Activation Test (COCAT), combining HaCaT keratinocytes with THP-1 cells as surrogate dendritic cells, was modified by incorporating a reconstructed human epidermis (RHE). The resulting 3D RHE/THP-1 coculture model was evaluated using chemicals that presented challenges for quantitative assessment in the 2D COCAT method. Results were compared with previously published 2D COCAT data and human and animal weight-of-evidence (WoE)-derived skin sensitization potency values (PVs). Incorporation of the RHE substantially expanded the applicability domain by enabling quantitative potency assessment of substances for which robust concentration–response relationships could not previously be established. The 3D RHE/THP-1 model differentiated all sixteen substances over more than three orders of magnitude in skin sensitization potency and enabled systematic comparison of structurally related substances. Alcohol–aldehyde potency relationships were highly structure dependent, ranging from minor differences between benzyl alcohol and benzaldehyde to approximately 15-fold differences between hydroxycitronellol and hydroxycitronellal. Potency estimates showed good overall agreement with published PVs. These findings demonstrate that incorporation of RHE extends the applicability domain of the COCAT method, provides a more physiologically relevant in vitro approach for quantitative skin sensitization potency assessment, and supports the further development of human-relevant, animal-free next-generation risk assessment strategies.

## Highlights

• RHE expands the applicability domain for difficult-to-test skin sensitizers.
• RHE/THP-1 model differentiates 16 chemicals over >3 orders of magnitude in potency.
• RHE enables quantitative assessment of fragrances and related substances.
• RHE/THP-1 agrees well with human skin sensitization potency estimates.
• RHE supports human-relevant, animal-free next-generation risk assessment.

## 1 Introduction

Skin sensitization is initiated when low-molecular-weight organic chemicals penetrate the skin and react with nucleophilic sites of endogenous proteins to form hapten–protein conjugates. In some cases, chemicals require metabolic activation (prohaptens) or abiotic activation (prehaptens) before becoming electrophilic and capable of protein binding (Aleksic and Meng, 2024). These events trigger a sequence of molecular and cellular responses that can ultimately culminate in allergic contact dermatitis (ACD), one of the most common inflammatory skin diseases in humans (Diepgen et al., 2015; Kaplan et al., 2012; Thyssen et al., 2007). This mechanistic understanding formed the basis for the OECD Adverse Outcome Pathway (AOP) for skin sensitization, which comprises four key events (KEs): covalent protein binding (KE1), keratinocyte activation (KE2), dendritic cell activation (KE3), and T-cell activation and proliferation (KE4) (OECD, 2014). The AOP has provided a robust mechanistic framework for the development of New Approach Methodologies (NAMs), most of which address one or more individual key events. The increasing implementation of animal-free Next-Generation Hazard and Risk Assessment requires NAMs that support hazard identification and quantitative skin sensitization potency assessment (Gilmour et al., 2023; Lee et al., 2026; OECD, 2025a; Vaillancourt et al., 2025). Quantitative potency estimates are essential for deriving points of departure for risk assessment and establishing safe exposure levels. Current in chemico and in vitro assays have proven highly successful for hazard identification within their respective applicability domains. Nevertheless, certain classes of substances, including those requiring metabolic or abiotic activation, highly lipophilic substances with low water solubility, or substances that induce excessive cytotoxicity under direct exposure conditions remain challenging to evaluate. These limitations are recognized in the applicability domains of several OECD test guidelines and highlight the need for complementary approaches capable of extending the chemical space accessible for quantitative potency assessment (OECD, 2024; OECD, 2025b; OECD, 2026).

One important difference between most conventional in vitro assays and the in vivo situation is the absence of a functional epidermal barrier. Following topical exposure, chemicals must initially penetrate the stratum corneum and diffuse through the viable epidermis, where their uptake, metabolism and interactions with keratinocytes and immune cells influence their subsequent biological activity. During this process, the epidermis regulates chemical penetration, modifies local exposure conditions, and provides a metabolically active tissue environment. Consequently, direct exposure of 2D-cultured cells cannot fully reproduce the physiological processes that influence the effective concentration of the ultimate reactive moiety reaching the responding cells. This limitation is expected to be particularly relevant for substances whose sensitization potency is influenced by penetration kinetics, tissue distribution, local metabolism, solubility and/or cytotoxicity.

The Cocultured Activation Test (COCAT) combines HaCaT keratinocytes with THP-1 cells, thereby integrating keratinocyte activation and dendritic cell activation within a single experimental system and functionally addressing two consecutive key events (KE2 and KE3) of the skin sensitization AOP. The method has proven suitable for hazard identification and quantitative potency assessment of a broad range of sensitizers (Eskes et al., 2019; Hennen et al., 2011; Hennen and Blömeke, 2017). However, a subset of substances remained difficult to evaluate due to limited aqueous solubility or insufficient responses within the experimentally accessible concentration range (Peter et al., 2023). Incorporation of a reconstructed human epidermis (RHE) into the COCAT offers the opportunity to combine topical exposure with a functional epidermal barrier (Schafer-Korting et al., 2008), thereby enabling the testing of substances with challenging physicochemical properties while maintaining the established THP-1 dendritic cell activation readout (OECD, 2024; Schellenberger et al., 2019). Rather than replacing the 2D COCAT method, the RHE/THP-1 approach was conceived as an extension of the existing method for substances that are difficult to evaluate under conventional submerged conditions.

The present study was designed to evaluate whether incorporation of a RHE extends the applicability domain of the 2D COCAT method. Rather than selecting a representative panel of only fragrance chemicals, the test set was intentionally assembled from substances that proved challenging in the quantitative evaluation of the 2D COCAT because of cytotoxicity, poor aqueous solubility or the inability to establish robust concentration-response relationships. Structurally related alcohol–aldehyde pairs and other analogues were included to investigate how incorporation of the RHE influences potency relationships among related substances. This approach was used to determine whether the barrier-containing RHE/THP-1 model expands the applicability domain for difficult-to-test substances while providing a more physiologically relevant basis for quantitative skin sensitization potency assessment.

## 2 Materials and methods

### 2.1 Chemicals and reagents

Anisyl alcohol (98%), α-amylcinnamic aldehyde (98.9%), α-amylcinnamyl alcohol, α-hexylcinnamic aldehyde (99.8%), benzyl benzoate (99.9%), benzyl salicylate (98%), cinnamic aldehyde (95%), cinnamyl alcohol (98%), 2-methoxy-4-methylphenol (100%), p-phenylenediamine (100%), and propidium iodide were purchased from Sigma-Aldrich (Taufkirchen, Germany). Benzaldehyde (99.8%), benzyl alcohol (100%), majantal (97.5%), majantol® (99.4%), hydroxycitronellal (99.8%), hydroxycitronellol (99.5%) were provided by Symrise AG (Holzminden, Germany). All chemicals were stored in amber HPLC vials under argon atmosphere until use. RPMI-1640 medium, fetal bovine serum (FBS), L-glutamine, sodium pyruvate, HEPES, phosphate-buffered saline (PBS), and antibiotic/antimycotic solution were obtained from Sigma-Aldrich. MycoAlert™ Mycoplasma Detection Kit was obtained from Lonza (Cologne, Germany). FITC-conjugated anti-human CD86 (clone 2331 [FUN-1]), APC-conjugated anti-human CD54 (clone 6.5B5), and mouse IgG1 isotype control antibody were obtained from BD Pharmingen (Heidelberg, Germany). Reconstructed human epidermis (SkinEthic^TM^ RHE, 0.5 cm²) models and culture medium were obtained from EPISKIN (Lyon, France).

### 2.2 Cultivation of THP-1 cells

The human monocytic THP-1 cell line was obtained from the German Collection of Microorganisms and Cell Cultures (DSMZ, Braunschweig, Germany). Cells were routinely tested and confirmed to be free of mycoplasma contamination using the MycoAlert™ Mycoplasma Detection Kit (Lonza, Cologne, Germany). Cell morphology and population doubling time were monitored regularly throughout the study. THP-1 cells (passages 4–15) were cultured in RPMI 1640 medium supplemented with 10% fetal bovine serum (FBS), 25 mM HEPES, 2 mM L-glutamine, 50 µM β-mercaptoethanol, and 1% antibiotic/antimycotic solution (100 U/mL penicillin, 100 µg/mL streptomycin, and 0.25 µg/mL amphotericin B) at 37°C in a humidified atmosphere containing 5% CO₂, as previously described (Hennen and Blömeke, 2017). Cells were maintained by routine passaging twice weekly and adjusted to a density of 0.2 × 10⁶ cells/mL for routine maintenance (Tsuchiya et al., 1980).

### 2.3 RHE/THP-1 coculture and topical exposure

The RHE/THP-1 coculture consists of a commercially available reconstructed human epidermis (RHE; SkinEthic™, EPISKIN, Lyon, France) combined with monocytic THP-1 cells as a surrogate for human dendritic cells. Following topical application of the test substance to the epidermal surface, the assay functionally integrates Key Events 2 and 3 within the OECD Adverse Outcome Pathway (AOP) for skin sensitization (OECD, 2025a), with dendritic cell activation serving as the assay readout. The SkinEthic™ RHE model is a highly differentiated, multilayered human epidermis reconstructed from primary human keratinocytes obtained from one to three donors. It comprises organized basal, spinous and granular cell layers together with a multilayered stratum corneum and closely reproduces the morphology and barrier properties of native human epidermis.

Cultivation of the RHE, establishment of the 3D RHE/THP-1 coculture, and chemical exposure were performed as previously described (Schellenberger et al., 2019). Briefly, 5 × 10⁵ THP-1 cells (cell viability ≥ 90%, determined by trypan blue exclusion) were seeded into the basolateral compartment of a 12-well plate in 500 µL phenol red-free RPMI 1640 medium supplemented with 10% FBS, 4 mM L-glutamine, and 25 mM HEPES. The RHE insert (surface area: 0.5 cm²) was then placed above the THP-1 cell suspension to establish the coculture. Test substances were diluted in acetone:olive oil (AOO; 4:1, v/v), and 6 µL was applied topically to the surface of each RHE (12 µL/cm²). The 3D RHE/THP-1 cocultures were incubated for 24 h at 37°C in a humidified atmosphere containing 5% CO₂. Unless otherwise indicated, test substances were initially evaluated at concentrations of 1%, 10%, and 30%. Additional concentrations were selected iteratively to refine the concentration–response relationship for each test substance.

### 2.4 CD86 and CD54 expression on cocultured THP-1 cells and positivity thresholds

After the incubation period, the RHE inserts were removed from the culture plates. THP-1 cells were harvested from the basolateral compartment, washed once with 2 mL phosphate-buffered saline (PBS), and centrifuged at 250 × g for 5 min. The cell pellet was resuspended in PBS, divided into appropriate aliquots, and stained separately with FITC-conjugated anti-human CD86 (clone 2331 [FUN-1]), anti-human CD54 (clone 6.5B5), or the corresponding mouse IgG1 isotype control. Cell surface marker expression was analyzed using a FACSVerse™ flow cytometer and BD FACSuite™ software (BD Biosciences, Heidelberg, Germany). Cell surface expression of CD86 and CD54 was determined on viable THP-1 cells (≥ 50%) after exclusion of propidium iodide (PI)-positive cells. Mean fluorescence intensity (MFI) values are converted to relative fluorescence intensity (RFI) by normalizing the MFI of exposed cells to that of the corresponding vehicle control after subtraction of the respective isotype control fluorescence according to the OECD Test Guideline 442E (OECD, 2024). The relevant concentration range for RHE exposure was established by assessing THP-1 cell viability using a propidium iodide exclusion assay. Concentrations resulting in a relative cell viability of at least 50% were selected for further testing. Experiments were repeated on several days using independent RHE tissues, different THP-1 cell passages, and freshly prepared test substance dilutions. Concentrations resulting in a relative cell viability of at least 50% were selected for further testing. Experiments were repeated on several days using independent RHE tissues, different THP-1 cell passages, and freshly prepared test substance dilutions. In accordance with the evaluation framework of OECD Test Guideline 442E, a positive response was defined as at least one activation marker reaching the predefined positivity threshold (CD86 ≥150% RFI or CD54 ≥200% RFI). A test substance was considered positive when the mean RFI calculated from the independent experiments reached the respective predefined threshold for either CD86 or CD54. In addition, at least two independent RHE tissues were required to exceed the respective threshold, thereby ensuring that classification was supported by reproducible biological responses rather than by the mean value alone. The lowest amount of test substance applied per unit area that fulfilled these criteria was defined as the RHE/THP-1 skin sensitization potency value (µg/cm²). To account for weaker responses, substances producing RFI values for either CD86 (RFI: 122–149%) or CD54 (RFI: 157–199%) were considered borderline in the present study.

### 2.5 Calculation of dose per unit area

The dose metric considered most relevant for the induction of skin sensitization is the amount of allergen applied per unit area of exposed skin, typically expressed as µg/cm² (Friedmann, 2007). Because the present assay involves topical application of a defined amount of test substance to the RHE surface, the applied concentrations can be converted to amounts per unit area (µg/cm²). A total volume of 6 µL test solution was applied to each RHE insert (surface area 0.5 cm²), corresponding to an application volume of 12 µL/cm². Depending on the concentration of test substance in the applied solution (1, 10, 30, or 100%, v/v), the corresponding nominal volumes of neat test substance were 0.12, 1.2, 3.6, and 12 µL/cm², respectively. For neat substances with densities close to 1 g/mL, these application volumes are approximately equivalent to 120, 1,200, 3,600 and 12,000 µg/cm². Exact amounts applied per unit area (µg/cm²) were calculated for each test substance using its respective density. The lowest amount applied per unit area that resulted in a positive assay response was used as the quantitative measure of skin sensitization potency (RHE/THP-1 potency value, µg/cm²).

### 2.6 Statistical analysis

RHE/THP-1 data are presented as mean ± SEM. The relationship between experimentally determined RHE/THP-1 potency values and published potency values (PVs) of the test substances was assessed. The PV represents the amount of a substance applied per unit area of skin at which skin sensitization is estimated to be induced and is expressed in µg/cm² (Irizar et al., 2025; Irizar et al., 2022). PVs were derived using a weight-of-evidence approach based on the best available human and/or experimental animal data, predominantly LLNA results, together with expert judgement, without incorporating in silico, in chemico, or in vitro data into the PV itself, thereby providing an independent benchmark for evaluating potency estimates generated by NAMs. Pearson's product-moment correlation and Spearman's rank correlation analyses were performed. For this analysis, potency values reported as exceeding the highest tested concentration (e.g., >10,188 or >25,000 µg/cm²) were conservatively represented by the corresponding threshold values. Correlation analyses were considered exploratory because of the limited number of test substances and the presence of censored observations. Statistical significance was defined as p < 0.05.

## 3 Results

An overview of the chemicals investigated in this study is presented in Table 1. The test set comprises two benchmark sensitizers together with structurally diverse fragrance and other substances spanning a broad range of physicochemical properties and structural classes. Most fragrance substances were selected because their quantitative evaluation in the 2D COCAT assay had previously been limited by excessive cytotoxicity, high lipophilicity, limited aqueous solubility, or the inability to establish robust concentration–response relationships (Peter et al., 2023). These substances (highlighted in bold in Table 1) were included to determine whether incorporation of a reconstructed human epidermis (RHE) expands the applicability domain of the assay. To provide reference points across a broad range of skin sensitization potencies, *p*-phenylenediamine and 2-methoxy-4-methylphenol were included as benchmark sensitizers. Hydroxycitronellol was additionally incorporated to extend the previous evaluation of the hydroxycitronellol/hydroxycitronellal alcohol–aldehyde pair to the 3D RHE/THP-1 approach. Overall, the selected chemicals span a wide range of lipophilicities (log K_ow_ −0.3 to 4.8) and structural classes, providing a suitable test set for evaluating the applicability domain of the RHE/THP-1 model and investigating structure-dependent skin sensitization potency relationships.

**Table 1:** Lipophilicity and structural classification of the test substances investigated in this study. The test set comprises reference sensitizers as well as fragrance and other substances spanning a broad range of lipophilicity. Log K_ow_ values (XLogP3) were obtained from the PubChem database. Bold text indicates substances for which testing issues were encountered in the conventional 2D COCAT method (Peter et al., 2023).

CAS Number	Chemical	Chemical structure	Log K_ow_ (XLogP3, PubChem)	Structural class
106-50-3	p-Phenylenediamine		-0.3	Aromatic diamine
93-51-6	2-Methoxy-4-methylphenol		1.3	Methoxy-substituted phenol
100-51-6	Benzyl alcohol		1.1	Aromatic alcohol
100-52-7	**Benzaldehyde**		1.5	Aromatic aldehyde
105-13-5	Anisyl alcohol		1.1	Aromatic alcohol
104-54-1	Cinnamyl alcohol		1.9	α,β-Unsaturated allylic alcohol
104-55-2	Cinnamic aldehyde		1.9	α,β-Unsaturated allylic aldehyde
107-74-4	Hydroxycitronellol		1.9	Acyclic monoterpene alcohol
107-75-5	Hydroxycitronellal		1.6	Acyclic monoterpene aldehyde
103694-68-4	**Majantol**		3.1	Branched aliphatic alcohol
107737-97-3	**Majantal**		2.9	Branched aliphatic aldehyde
101-85-9	**α-Amylcinnamyl alcohol**		4.4	Lipophilic α,β-unsaturated allylic alcohol
122-40-7	**α-Amylcinnamic aldehyde**		4.2	Lipophilic α,β-unsaturated allylic aldehyde
101-86-0	**α-Hexylcinnamic aldehyde**		4.8	Lipophilic α,β-unsaturated allylic aldehyde
120-51-4	**Benzyl benzoate**		4.0	Aromatic ester
118-58-1	**Benzyl salicylate**		3.2	Aromatic ester

Fig. 1 illustrates the experimental workflow and biological concept of the 3D RHE/THP-1 coculture model. A commercially available reconstructed human epidermis (SkinEthic™ RHE) cultured on a porous Transwell membrane was combined with THP-1 cells in the basolateral compartment. During topical exposure, the RHE provides a physiologically relevant epidermal barrier while retaining the metabolic competence of differentiated keratinocytes. Consequently, THP-1 cells may be exposed to test substances and their transformation products that traverse the epidermis, as well as to soluble mediators released by the tissue in response to chemical exposure (Wang et al., 2025). After 24 h of coculture, THP-1 cells were harvested and analyzed for viability and expression of the dendritic cell activation markers CD86 and CD54 by flow cytometry.

**Fig. 1:**
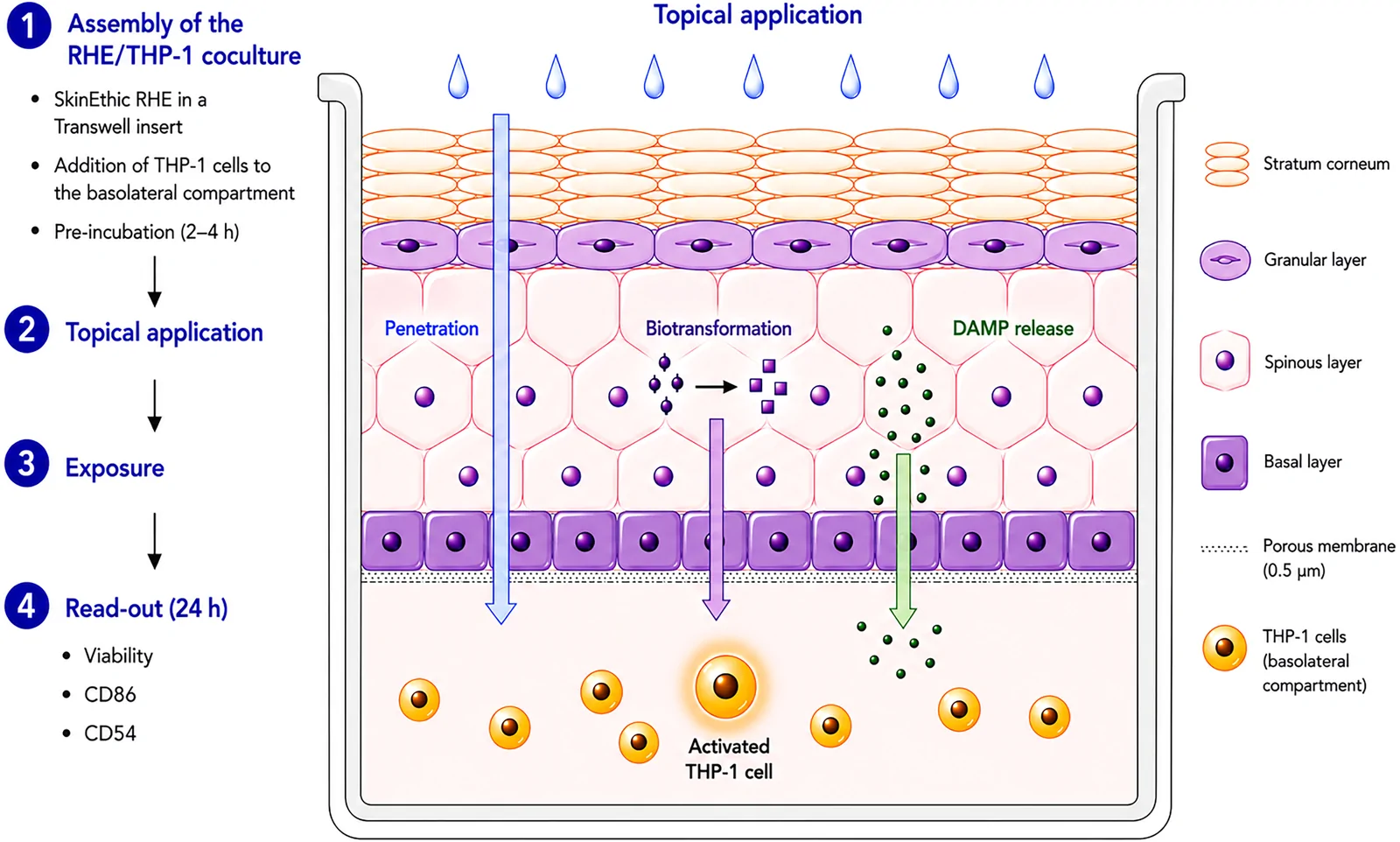
Graphical summary of the biological concept underlying the 3D RHE/THP-1 coculture model. Commercially available reconstructed human epidermis (RHE; SkinEthic™ RHE) cultured on a porous Transwell membrane was combined with THP-1 cells in the basolateral compartment and pre-incubated prior to topical application of the test substances. Following topical application, the THP-1 response may reflect the combined influence of penetration of the parent substance through the epidermal barrier, metabolic transformation of the parent substance within viable keratinocytes, and the release of damage-associated molecular patterns (DAMPs), cytokines, and other soluble mediators. After 24 h of exposure, THP-1 cells were harvested for determination of cell viability and analysis of CD86 and CD54 expression by flow cytometry.

### 3.1 Expansion of the applicability domain by integration of reconstructed human epidermis

Integration of RHE expanded the applicability domain of the assay for challenging fragrance chemicals (Table 2). In particular, the highly lipophilic fragrance pair α-amylcinnamyl alcohol/α-amylcinnamic aldehyde, the aromatic aldehyde benzaldehyde, and the fragrance substance majantol with its structurally related aldehyde majantal, which could not be quantitatively evaluated in the conventional 2D COCAT, generated reproducible dendritic cell activation responses in the 3D RHE/THP-1 model. Likewise, the esters benzyl benzoate and benzyl salicylate, which had previously challenged quantitative assessment because of their high lipophilicity and limited aqueous solubility, could be evaluated following topical application to the reconstructed epidermis.

**Table 2:** Dendritic cell activation and skin sensitization potency of fragrance and other substances in the RHE/THP-1 model and comparison with published potency values (PVs). Relative fluorescence intensities (RFI, %, mean ± SEM) of CD86 and CD54 expression on THP-1 cells after 24 h of topical exposure of reconstructed human epidermis (RHE) to the indicated test substances. THP-1 viability (%) at the RHE/THP-1 potency value is shown. The RHE/THP-1 potency value represents the lowest concentration applied to the RHE surface (µg/cm²) that elicited a positive response according to the predefined evaluation criteria. Published PVs represent skin sensitization induction thresholds derived using a weight-of-evidence approach based on the best available human and/or experimental animal data (Irizar et al., 2025; Irizar et al., 2022). Substances not assigned a numerical PV are indicated as *NA* or according to the published classification.

Chemical	RFI (%) on cocultured THP-1 of RHE/THP-1 assay				Viability of THP-1 (%) at potency value	3D RHE/THP-1 potency value (µg/cm²)	Published WoE Potency Value (PV) (µg/cm²)^1^
	CD86	SEM	CD54	SEM			
p-Phenylenediamine	243	123	379	84	93	18	3.9
2-Methoxy-4-methylphenol	283	71	406	133	82	166	1,350
Benzyl alcohol	180	40	89	32	83	1,298	>25,000
Benzaldehyde	185	75	135	79	93	1,019	4,094
Anisyl alcohol	155	10	85	11	70	1,658	1,475
Cinnamyl alcohol	151	11	192	45	69	322	5,775
Cinnamic aldehyde	169	12	142	11	71	159	885
Hydroxycitronellol	159	41	166	32	93	1,255	*NA*
Hydroxycitronellal	432	133	1,516	468	89	83	5,275
Majantol	169	58	158	24	82	107	16,250
Majantal	253	77	102	21	65	211	*NA*
α-Amylcinnamyl alcohol	177	30	177	30	70	245	very weak/non-sensitizer
α-Amylcinnamic aldehyde	249	47	189	42	68	485	23,622
α-Hexylcinnamic aldehyde	216	59	150	37	66	2,077	23,620
Benzyl benzoate	114	33	127	19	100	>10,188	>25,000
Benzyl salicylate	114	4	157	5	94	*4,108*	17,715

1 The PV represents the amount of a substance applied per unit area of skin at which skin sensitization is estimated to be induced. PVs were derived using a weight-of-evidence approach based on the best available human and/or experimental animal data, together with expert judgement, without incorporating in silico, in chemico, or in vitro data into the PV itself, thereby providing an independent benchmark for evaluating potency estimates generated by NAMs (Irizar et al., 2025; Irizar et al., 2022).

RHE/THP-1 potency values or borderline values were obtained for all investigated chemicals at concentrations maintaining acceptable THP-1 viability (mean 78.5%; range 66–100%). As expected, the reference sensitizer p-phenylenediamine reached the lowest RHE/THP-1 potency value (18 µg/cm²), whereas substantially higher amounts were required for the other chemicals. Among the positively classified chemicals, α-hexylcinnamic aldehyde required the highest topical dose (2,077 µg/cm²). Of the ester-containing chemicals, benzyl salicylate produced a borderline response at 4,108 µg/cm², whereas benzyl benzoate remained negative throughout the tested concentration range, including the highest applied dose (>10,188 µg/cm²). Overall, the RHE/THP-1 assay enabled quantitative assessment of chemicals spanning more than three orders of magnitude in skin sensitization potency, including several substances that could not previously be quantitatively evaluated in the conventional 2D COCAT. The experimentally determined RHE/THP-1 potency values were compared with published human weight-of-evidence potency values (PVs). The RHE/THP-1 model covered the broad spectrum of skin sensitization potencies while enabling quantitative evaluation of substances that could not be reliably assessed in the conventional 2D COCAT method (Table 2). Following log₁₀ transformation, the RHE/THP-1 potency values showed a significant positive correlation with the PVs (Pearson’s *r* = 0.678, *p* = 0.011; Spearman’s ρ = 0.633, *p* = 0.020; *n* = 13). For substances with PVs reported above the highest tested concentration, the corresponding threshold value was used as a conservative lower-bound estimate for the correlation analysis. These correlations should be interpreted cautiously because of the limited number of substances, the presence of censored observations, and the fact that the published PVs are weight-of-evidence estimates rather than direct experimental measurements.

### 3.3 Structure-dependent alcohol–aldehyde potency relationships in the RHE/THP-1 model

Table 3 compares the RHE/THP-1 potency values (µg/cm²) determined for the investigated alcohol–aldehyde pairs with the previously published 2D COCAT potency estimates (Peter et al., 2023). Whereas several substances could not be quantitatively evaluated in the 2D COCAT method, incorporation of RHE enabled direct comparison of all investigated alcohol–aldehyde pairs in the 3D RHE/THP-1 model. The observed potency relationships differed substantially among the structural classes investigated. The aromatic fragrance alcohol benzyl alcohol exhibited only weak activity in the conventional 2D COCAT, yielding an ECΔ value of 3.80 mM. Under the same experimental conditions, benzaldehyde did not induce a positive response, precluding direct comparison of the benzyl alcohol–benzaldehyde pair within the 2D COCAT. In contrast, both chemicals elicited reproducible positive responses in the RHE/THP-1 model. This aromatic alcohol–aldehyde pair exhibited the smallest difference in potency, with RHE/THP-1 potency values differing by only 1.3-fold (1,298 and 1,019 µg/cm², respectively). Anisyl alcohol elicited a similar positive response at 1,658 µg/cm² (Table 2). Although the corresponding aldehyde (anisaldehyde) was not available for testing, these findings indicate only minor potency differences among the investigated aromatic fragrance alcohols and benzaldehyde.

**Table 3:** Comparison of structurally related alcohols and aldehydes based on published COCAT data and results from the 3D RHE/THP-1 model. COCAT ECΔ values (mM) represent the lowest concentration inducing a positive response in the COCAT method, as reported (Peter et al., 2023). The 3D RHE/THP-1 potency values represent the lowest amount applied to the RHE surface (µg/cm²) that elicited a positive response, defined as CD86 ≥150% RFI and/or CD54 ≥200% RFI according to the predefined evaluation criteria. Alcohol-to-aldehyde potency ratios were calculated from the 3D RHE/THP-1 potency values, and the more potent substance within each pair is indicated.

Alcohol	Aldehyde	COCAT ECΔ (mM)^2^	3D RHE/THP-1 potency value (µg/cm²)	Alcohol/aldehyde potency ratio (3D RHE/THP-1)	More potent chemical
Benzyl alcohol	Benzaldehyde	3.80 vs >4,000^3^	1,298 vs 1,019	1.3	Benzaldehyde
Cinnamyl alcohol	Cinnamic aldehyde	0.88 vs 0.14	322 vs 159	2.0	Cinnamic aldehyde
Hydroxycitronellol	Hydroxycitronellal	1.6 vs 0.15	1,255 vs 83	15	Hydroxycitronellal
Majantol	Majantal	Not testable (solubility)	107 vs 211	0.5	Majantol
α-Amylcinnamyl alcohol	α-Amylcinnamic aldehyde	Not testable (cytotoxicity)	245 vs 485	0.5	α-Amylcinnamyl alcohol

^2^Data taken from Peter et al. (2023). ECΔ represents the lowest concentration eliciting a positive response in the COCAT assay.

^3^(not comparable)

For the cinnamyl alcohol and cinnamic aldehyde pair, both substances elicited reproducible responses in the 2D COCAT and 3D RHE/THP-1 assays. In the 2D COCAT, cinnamic aldehyde exhibited an approximately six-fold higher potency than cinnamyl alcohol (Peter et al., 2023). In the 3D RHE/THP-1 model, this difference decreased to approximately two-fold. Cinnamic aldehyde reached an RHE/THP-1 potency value of 159 µg/cm², whereas cinnamyl alcohol required 322 µg/cm². The fragrance substance majantol and the structurally related aldehyde majantal also exhibited similar activities in the 3D RHE/THP-1 assay. Both substances, which could not be quantitatively evaluated in the conventional 2D COCAT, generated reproducible positive responses with 3D RHE/THP-1 potency values of 107 and 211 µg/cm², respectively, corresponding to an approximately two-fold higher potency of majantol. A comparable relationship was observed for the α-amyl derivatives. Although both substances had previously inconclusive assessments in the 2D COCAT, α-amylcinnamyl alcohol could be conclusively evaluated in the 3D RHE/THP-1 assay and reached an RHE/THP-1 potency value of 245 µg/cm², whereas α-amylcinnamic aldehyde required 485 µg/cm², corresponding to an approximately two-fold higher potency of the alcohol. In contrast, hydroxycitronellol and hydroxycitronellal exhibited markedly different potencies. Hydroxycitronellal reached an RHE/THP-1 potency value of 83 µg/cm², whereas hydroxycitronellol required 1,255 µg/cm², indicating that hydroxycitronellal was approximately 15-fold more potent than hydroxycitronellol.

Taken together, the RHE/THP-1 model enabled quantitative comparison of structurally related alcohol–aldehyde pairs across a broad range of skin sensitization potencies, demonstrating that potency relationships between alcohols and their corresponding aldehydes are highly structure dependent.

## 4 Discussion

The present study demonstrates that incorporation of a reconstructed human epidermis (RHE) substantially expands the applicability domain of dendritic cell activation assays, contributing substantially more than just being a physical barrier to chemical penetration. Following topical application, chemicals penetrate the stratum corneum and viable epidermis, where they are subjected to the combined influence of epidermal architecture, metabolic competence, and keratinocyte responses before reaching the underlying THP-1 cells. Simultaneously, chemical exposure and cellular stress induce the release of danger-associated molecular patterns (DAMPs), cytokines, and other soluble mediators that contribute to activation of the underlying THP-1 cells (Fig. 1). Although the 3D RHE/THP-1 assay measures Key Event 3 (dendritic cell activation) of the OECD AOP for skin sensitization, incorporation of a RHE enables this key event to be assessed in the context of biologically relevant upstream epidermal processes.

The principal finding of the present study is that incorporation of a RHE expands the applicability domain of dendritic cell activation assays for fragrance and other substances that are difficult to evaluate under conventional in vitro conditions. Monolayer-based dendritic cell activation assays have proven highly successful for skin sensitization hazard identification and are widely used within integrated testing strategies. However, certain classes of chemicals, including highly lipophilic substances, poorly water-soluble substances, and chemicals causing pronounced cytotoxicity under direct exposure conditions, remain challenging to evaluate quantitatively because robust concentration–response relationships cannot always be established within the biologically relevant concentration range (Peter et al., 2023). Introducing a differentiated RHE fundamentally changed the biological context of chemical exposure. Instead of direct addition to submerged cell cultures, test substances were applied topically to a functional stratum corneum, allowing them to penetrate the viable epidermis before reaching the underlying THP-1 compartment. This barrier-controlled exposure more closely resembles dermal exposure in humans and enabled quantitative assessment of fragrance chemicals that had previously been difficult or impossible to evaluate in the 2D COCAT (Table 2, Table 3). Importantly, the performance of the 3D RHE/THP-1 model was not reflected by a general increase or decrease in assay sensitivity but by the ability to establish robust concentration–response relationships over an expanded range of chemicals. This was particularly evident for the highly lipophilic α-amyl derivatives, α-hexylcinnamic aldehyde, benzaldehyde, the structurally related pair majantol and majantal, and the ester-containing substances benzyl benzoate and benzyl salicylate, all of which could be quantitatively evaluated in the 3D RHE/THP-1 model. Together, these observations demonstrate that incorporation of a physiologically relevant epidermal barrier substantially extends the applicability domain beyond that achievable with conventional submerged culture systems. However, although RHE models reproduce key structural and functional features of the epidermal barrier, their barrier function does not fully reproduce that of native human skin and may result in substance-dependent differences in penetration (Bouwstra et al., 2021; Hagvall et al., 2023). The improved performance of the RHE/THP-1 model is likely to result from several characteristics of reconstructed human epidermis beyond barrier-controlled exposure conditions. Compared with the HaCaT monolayer used in the COCAT method, reconstructed epidermis more closely reproduces the architecture of human skin and retains a broader spectrum of epidermal metabolic competence, including enzymes involved in both chemical activation and detoxification (Eilstein et al., 2015; Eilstein et al., 2014). In addition, topical exposure of differentiated keratinocytes has been shown to induce the release of DAMPs, cytokines, chemokines, and other soluble mediators capable of modulating dendritic cell activation (Hennen et al., 2011; Klicznik et al., 2018; Nestle et al., 2009). Although these processes were not investigated directly in the present study, they represent plausible biological mechanisms contributing to the improved performance of the RHE/THP-1 model. Consequently, the THP-1 response likely reflects the integrated influence of barrier-controlled exposure, epidermal metabolic competence, and keratinocyte-derived signaling, including the effects of parent substances and their transformation products. Although the relative contribution of these individual processes remains to be established, their integration within a single experimental system provides a more physiologically relevant framework for quantitative skin sensitization potency assessment. The complementary nature of CD86 and CD54 responses should also be considered when interpreting the THP-1 readout. Individual test substances showed different response patterns, with responses exceeding the respective threshold for CD86, CD54, or both markers (Table 2). Requiring both markers to exceed their respective thresholds would therefore have resulted in substantially fewer positive classifications in the present dataset. This observation supports the predefined either-marker criterion, which follows the h-CLAT evaluation framework, where CD86 and CD54 responses are considered without requiring both markers to exceed their respective thresholds.

One objective of the present study was to investigate whether incorporation of an RHE influences skin sensitization potency relationships among structurally related alcohols and aldehydes. Comparison of representative alcohol–aldehyde pairs revealed markedly different potency relationships, ranging from almost identical activities to differences exceeding one order of magnitude (see Table 3). These findings demonstrate that alcohol–aldehyde potency relationships cannot be generalized across the studied substances but are structure dependent. Whereas for most pairs of aldehydes and alcohols the potency differed maximum by a factor of 2, hydroxycitronellol and hydroxycitronellal differed by approximately 15-fold. It is noteworthy to say that for these selected pairs of aldehydes and alcohols, the aldehydes were not constantly more potent than the alcohols, as e.g. α-amylcinnamyl alcohol proved approximately 2-fold more potent than its corresponding aldehyde. The hydroxycitronellol/hydroxycitronellal pair provides a particularly illustrative example. While both substances were identified as sensitizers in both systems, the RHE/THP-1 model differentiated their potencies by approximately 15-fold. This observation further indicates that incorporation of the RHE not only enabled quantitative assessment of both substances but also improved resolution between structurally related chemicals with markedly different human sensitization potencies. The mechanisms underlying these structure-dependent potency relationships were not investigated in the present study. Nevertheless, the findings suggest that the skin sensitization potency measured in the 3D RHE/THP-1 model reflects the integrated response of a differentiated epidermis rather than a single biological process (see Fig. 1). Barrier-controlled chemical penetration, epidermal biotransformation, and keratinocyte-derived signaling are all likely to contribute to the observed responses, although their relative importance probably varies between chemical classes. Further mechanistic studies will be required to define the relative contribution of these processes to quantitative skin sensitization potency assessment.

Alcohol oxidation to the corresponding aldehydes has long been considered the principal activation pathway in the epidermis and has frequently been proposed to explain differences in skin sensitization potency between structurally related alcohols and aldehydes (Aleksic et al., 2024; Eilstein et al., 2014). In addition to enzymatic oxidation, cinnamyl alcohol can undergo rapid autoxidation upon air exposure, generating oxidation products including cinnamic aldehyde, epoxy cinnamyl alcohol, and cinnamic acid (Niklasson et al., 2013). If alcohol oxidation to the corresponding aldehyde were the dominant determinant of measured skin sensitization potency, structurally related alcohol–aldehyde pairs might be expected to exhibit more similar biological activities than observed in the present study. The relatively small potency difference between benzyl alcohol and benzaldehyde is consistent with oxidation contributing to the activity of this substance pair. In contrast, the pronounced difference between hydroxycitronellol and hydroxycitronellal indicates that oxidation alone is unlikely to account for the observed potency. Collectively, these observations suggest that the contribution of alcohol oxidation to skin sensitization potency is structure dependent. In addition to alcohol oxidation, alternative metabolic pathways may contribute to the skin sensitization potency of selected alcohols. Aromatic alcohols containing a benzylic or allylic hydroxyl group, including benzyl alcohol, anisyl alcohol and cinnamyl alcohol, have been shown to undergo sulfotransferase (SULT)-mediated activation in the epidermis, generating reactive sulfate esters capable of protein binding (Moss et al., 2016; Natsch et al., 2018). As reconstructed human epidermis expresses functional sulfotransferases (Eilstein et al., 2015; Eilstein et al., 2014), this pathway may contribute to the potency of these alcohols. Likewise, benzyl benzoate and benzyl salicylate (SCCS, 2023) are expected to undergo esterase-mediated hydrolysis in the epidermis (Telaprolu et al., 2023), yielding benzyl alcohol as a common hydrolysis product, consistent with the documented esterase activity of reconstructed human epidermis and human tissues (Eilstein et al., 2015; Eilstein et al., 2014). However, the different responses of the two esters in the present study indicate that formation of benzyl alcohol alone is insufficient to explain the results, suggesting that additional substance-specific processes influence the overall biological response. Collectively, these observations indicate that no single metabolic activation pathway is sufficient to explain the diverse potency relationships among structurally related chemicals. The complexity of the observed potency relationships is consistent with published clinical and toxicological evidence showing that structurally related fragrance alcohols differ considerably in their human skin sensitization potency, suggesting that alcohol-to-aldehyde conversion alone is insufficient to explain their sensitization behavior (Api et al., 2015; Karlberg et al., 2013). Benzyl alcohol, for example, is generally regarded as a weak human skin sensitizer, while LLNA studies have reported variable but overall low sensitization activity (Basketter et al., 2014). It has recently been designated the American Contact Dermatitis Society’s *Allergen of the Year 2026*, reflecting its increasing recognition as an underestimated cause of allergic contact dermatitis (Geier et al., 2022; Le and Wu, 2026). In contrast to the result in the 2D COCAT method, where benzaldehyde proved negative, the incorporation of the reconstructed epidermis yielded a result more in line with the human situation (Irizar et al., 2022). Comparison of the experimentally determined RHE/THP-1 potency values with the published potency values (PVs) (Irizar et al., 2025; Irizar et al., 2022) showed good overall concordance. The RHE/THP-1 model covered the broad spectrum of skin sensitization potencies while substantially extending the range of substances that could be quantitatively evaluated (Table 2). The observed significant correlation further supports the consistency of the RHE/THP-1 potency estimates with the published PVs. However, these findings should be interpreted cautiously because of the limited number of substances, the presence of censored observations, and the fact that PVs are weight-of-evidence estimates derived from human and/or experimental animal data rather than direct experimental measurements. Nevertheless, the overall concordance suggests that the RHE/THP-1 model broadly captures the human skin sensitization potency spectrum while enabling quantitative assessment of substances that are difficult to evaluate under conventional submerged culture conditions.

In conclusion, the present findings demonstrate that incorporation of a RHE substantially extends the applicability domain of dendritic cell activation assays for fragrance and other difficult-to-test substances. By combining topical exposure with a differentiated human epidermis, the 3D RHE/THP-1 model complements existing skin sensitization NAMs and enables quantitative evaluation of substances that cannot be reliably assessed under conventional submerged culture conditions. Although the present evaluation was limited to several fragrance and other substances, the overall agreement with published human weight-of-evidence potency estimates is encouraging and supports further evaluation of the model. Such studies will help further define its applicability domain and clarify its contribution to integrated animal-free next-generation risk assessment (Gilmour et al., 2023; OECD, 2025a).

## CRediT authorship contribution statement

**Brunhilde Blömeke:** Writing – review & editing, Writing – original draft, Visualization, Supervision, Project administration, Funding acquisition, Formal analysis, Conceptualization. **Niklas Peter:** Writing – review & editing, Methodology, Formal analysis, Data curation. **Udo Bock:** Writing – review & editing, Methodology, Funding acquisition, Formal analysis, Conceptualization. **Boris Peter Müller:** Writing – review & editing, Conceptualization. **Peter Griem:** Writing – review & editing, Methodology, Formal analysis, Conceptualization. **Rüdiger Wittlake:** Writing – review & editing, Conceptualization. **Johannes Panten:** Writing – review & editing, Conceptualization.

## Declaration of competing interest

The authors declare that they have no known competing financial interests or personal relationships that could have appeared to influence the work reported in this paper.

## Data Availability

Data will be made available on request.
